# Severe upper airway obstruction due to delayed retropharyngeal hematoma formation following blunt cervical trauma

**DOI:** 10.1186/1471-2253-7-2

**Published:** 2007-03-12

**Authors:** Laurie W Lazott, John A Ponzo, Rudolph B Puana, Katie S Artz, David P Ciceri, William C Culp

**Affiliations:** 1 Department of Anesthesiology, The Texas A&M University System Health Science Center College of Medicine, Scott & White Hospital, Temple, Texas, USA; 2 Department of Radiology, The Texas A&M University System Health Science Center College of Medicine, Scott & White Hospital, Temple, Texas, USA; 3 Department of Surgery, The Texas A&M University System Health Science Center College of Medicine, Scott & White Hospital, Temple, Texas, USA

## Abstract

**Background:**

We report a case of severe upper airway obstruction due to a retropharyngeal hematoma that presented nearly one day after a precipitating traumatic injury. Retropharyngeal hematomas are rare, but may cause life-threatening airway compromise.

**Case presentation:**

A 50 year-old man developed severe dyspnea with oropharyngeal airway compression due to retropharyngeal hematoma 20 hours after presenting to the emergency department. The patient also had a fractured first cervical vertebra and was diagnosed with a left brachial plexopathy. The patient underwent emergent awake fiberoptic endotracheal intubation to provide a definitive airway.

**Conclusion:**

Retropharyngeal hematoma with life-threatening airway compromise can develop hours or days after a precipitating injury. Clinicians should be alert to the potential for this delayed airway collapse, and should also be prepared to rapidly secure the airway in this patient population likely to have concomitant cervical spinal or head injuries.

## Background

Retropharyngeal hematomas large enough to cause severe upper airway obstruction and respiratory distress are rare. Hematoma development and symptom onset may be delayed from an injury and as a result, initial diagnostic radiographic studies may be unremarkable. Treatment in the dyspneic patient should include immediate stabilization of the airway because initial symptoms may progress rapidly to severe upper airway obstruction and death. We describe a patient who developed a retropharyngeal hematoma that caused acute airway compromise 20 hours after presenting to the emergency department with a fracture of the first cervical vertebra. The rationale for our management strategy utilizing an awake fiberoptic intubation technique is also detailed.

## Case presentation

**Figure 1:**
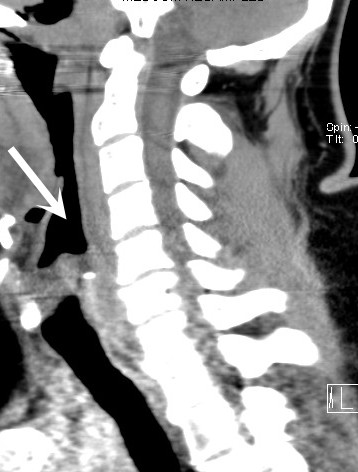
Sagittal reconstruction from a CT scan upon patient arrival shows mild prominence of the prevertebral soft tissues, without clinically significant hematoma or encroachment on the airway (large white arrow).

**Figure 2:**
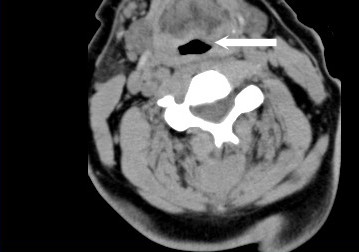
Axial reconstruction from a CT scan upon patient arrival show mild prominence of the prevertebral soft tissues, without clinically significant hematoma or encroachment on the airway (large white arrow).

A 50-year-old man presented to the emergency department after sustaining blunt trauma to the head secondary to a fall from a horse. The patient's forehead struck the ground with his neck extended. The patient complained of neck pain and left arm weakness and paresthesias, and denied loss of consciousness. The patient's past medical history was otherwise unremarkable, and did not include use of anticoagulants or anti-platelet agents. Laboratory studies, including coagulation profile and platelet count, were within normal limits both immediately after admission and on the following day. A rigid cervical collar was applied upon arrival to the emergency department. The patient's vital signs were stable and he was treated conservatively with intravenous maintenance fluid and opioid medication for pain control. The patient received no anticoagulation therapy (specifically no low molecular weight heparin), no platelet inhibitors or non-steroidal anti-inflammatory medications and no treatment modalities which could have potentially contributed to a coagulation disorder. Initial computed axial tomography (CT) of the cervical spine revealed bilateral fractures of the anterior arch of C1 and a fracture of the right C4 spinous process. Mild prominence of the prevertebral soft tissues was noted without significant encroachment on the pharynx (Figures 1, 2).

**Figure 3:**
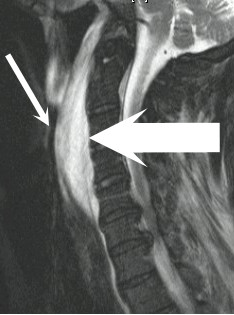
Sagittal T2 weighted MRI with fat saturation techniques revealing marked increase in the prevertebral soft tissue prominence secondary to an enlarging hematoma (large arrow). Note the severe compromise of the oropharyngeal airway (small arrow).

Approximately 20 hours after his initial presentation, the patient underwent magnetic resonance imaging (MRI) of the cervical spine to further evaluate his upper extremity neurologic deficits. While undergoing MRI, the patient developed sudden dyspnea and hoarseness. MRI demonstrated development of a marked increase in the prevertebral soft tissue prominence due to an enlarging retropharyngeal hematoma. Severe compromise of the oropharyngeal airway was evident (Fig. 3).

The patient was treated with oxygen at the MRI suite while members of both the surgery and anesthesiology department were contacted for emergent management. Upon their arrival, the patient had a blood pressure of 150/80 mmHg, pulse of 66 beats per minute, a 99% oxygen saturation level, respiratory rate of 24 breaths per minute, and appeared in mild distress. He was immediately transported to the intensive care unit by the physician team for definitive airway control. The patient was prepared for awake nasal fiberoptic intubation with intravenous glycopyrrolate and lidocaine spray topically to the nasopharynx. The bronchoscope was passed through the nasopharynx, vocal cords, and into the trachea. The pharynx and superior trachea were severely distorted and compressed. A 7.0 mm endotracheal tube was passed over the bronchoscope and positioned approximately 3 cm above the carina. The patient was comfortable and did not move during the procedure. Cervical spine immobilization was maintained throughout. Additionally, a surgeon and instruments were present throughout the procedure in anticipation of emergent tracheostomy if fiberoptic intubation were unsuccessful. After intubation, the patient was sedated and mechanically ventilated.

**Figure 4:**
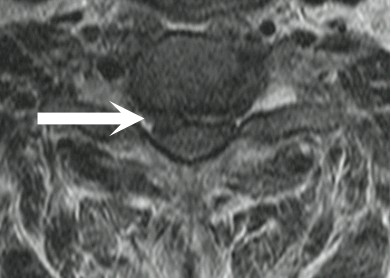
Axial T2 weighted MRI reveals a bi-lobed disc herniation (arrow), with secondary spinal canal encroachment and mass effect on the cervical spinal cord at the C6-7 level.

**Figure 5:**
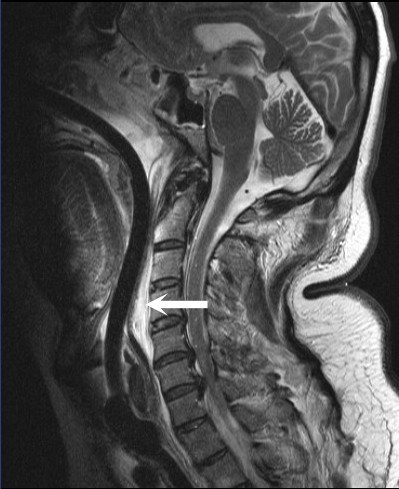
After surgical decompression, hematoma size is demonstrably reduced (arrow) in this sagittal MRI.

The following day the patient underwent surgical decompression. A moderate sized hematoma at the level of C4 was evacuated and a drain was placed for further decompression. A follow-up MRI was performed which revealed a bi-lobed disc herniation (determined to be chronic in nature) with secondary spinal canal encroachment and mass effect on the cervical spinal cord (Figures 4, 5). The retropharyngeal hematoma had resolved. The patient was successfully extubated with the use of a Cook exchange catheter on post-operative day one and discharged two days later.

## Discussion

The retropharyngeal space is a potential space that lies posterior to the buccopharyngeal fascia surrounding the pharynx, anterior to the prevertebral fascia of the cervical and thoracic spine and extends laterally to the carotid sheaths. It begins at the base of the skull and terminates in the superior mediastinum [1,2]. The mechanisms triggering hemorrhage into the retropharyngeal space are thought to include injury to the longus colli muscles on the anterior surface of the vertebral bodies, the anterior longitudinal ligament or the anterior muscular and spinal branches of the vertebral arteries [2-5]. These injuries are most commonly associated with closed cervical neck trauma [6] and anticoagulant therapy [1], but they have also been attributed to blunt head trauma, bleeding diathesis, cannulation of the internal jugular vein, arteriography, whiplash injury, foreign body ingestion, retropharyngeal infection, carotid artery aneurysm, carotid sinus massage, metastatic disease and can even occur spontaneously [6-8].

As blood enters the retropharyngeal space, the expanding hematoma can cause tracheal compression, which may rapidly progress to acute airway obstruction [1,3]. The incidence of airway obstruction resulting from retropharyngeal hematomas is low, but its occurrence can be life threatening [2,4,7].

Patients classically present with "Capp's Triad" which includes compression of the trachea and esophagus, displacement of the trachea anteriorly and bruising of the neck and chest [8]. Our patient had minimal bruising, but did demonstrate tracheal compression and displacement. The initial symptoms of airway compression may include dyspnea, dysphagia, stridor, odynophagia, hoarseness and neck pain [6,9]. Patients whose symptoms are suggestive of retropharyngeal hematomas warrant close and often prolonged follow-up because, as with our patient, a delay can exist between the patient's initial injury or symptoms and the development of respiratory distress [1]. Although most patients become symptomatic immediately or within hours of the development of the hematoma, there have been reports of patients who experienced airway obstruction as long as 5 days after developing the initial symptoms of airway compression [4,6]. Clinicians should maintain a high degree of suspicion when evaluating patients presenting with symptoms of airway compression whose mechanism of injury is consistent with those associated with retropharyngeal hematomas because these initial symptoms may progress rapidly to lethal airway obstruction. In particular, dyspnea should be considered a key clinical indicator of a possible airway crisis because significant airway compression must occur prior to a healthy patient reporting a dyspneic sensation [3].

Although treatment course may vary depending on the etiology of the hematoma, the first step in management is establishing definitive airway control [5]. If no airway compromise is present and the patient has a small, stable hematoma, conservative management and observation are indicated. These patients should be followed radiographically to ensure complete resolution of the hematoma [4,5]. Patients with airway compromise, however, should be managed aggressively utilizing a multidisciplinary approach including an experienced anesthesiologist and surgeon prepared to perform emergent tracheostomy if indicated. Careful attention should be directed towards any associated spinal injuries which may complicate airway management, making a difficult intubation even more challenging.

We chose an awake nasal fiberoptic intubation technique to secure our patient's airway. This avoided the risk of airway collapse resulting from traditional intravenous induction agents, although ketamine, coupled with an antisialogogue, would have been a viable alternative because it exerts minimal effects on muscular airway tone [10]. Furthermore, this approach allowed direct visualization of the patient's airway anatomy with negligible manipulation of the hematoma [1,5]. We were careful to limit our manipulation as much as possible, for fear of hematoma rupture with resultant bleeding and worsening airway compromise [5]. The fiberoptic approach allowed us to maintain cervical spine alignment during intubation minimizing risk of further spinal injury.

## Conclusion

Retropharyngeal hematoma is a rare disease, but may develop distant from a presenting injury and in the setting of normal coagulation and platelet function. Resultant dyspnea is an ominous sign of impending respiratory collapse and requires promptly securing the airway. Concomitant spinal injuries should be sought and when present, may further complicate attempts at airway management. We encourage close and prolonged follow up both clinically and radiographically of these patients, and would consider sequential CT scans in high risk groups. We advocate awake fiberoptic intubation with surgical backup as an optimal approach for managing these patients.

## Competing interests

The author(s) declare that they have no competing interests.

## Authors' contributions

Each of the authors cared for the patient. JAP prepared the figures; LWL and WCC drafted the manuscript. Each of the authors has read and approved the manuscript.

## Pre-publication history

The pre-publication history for this paper can be accessed here:


